# Use of Promethazine and Meperidine as Premedicants in Asthmatics before Initiation of Surgery: An Interim Report

**DOI:** 10.22114/ajem.v0i0.229

**Published:** 2019-07-29

**Authors:** Zahid Hussain Khan, Milad Minagar, Mohammad Dehghan-Tezerjani

**Affiliations:** 1. Department of Anesthesiology and Critical Care, Imam Khomeini Medical Complex, Tehran University of Medical Sciences, Tehran, Iran.

Patients with bronchial asthma usually land up for surgery and pose a significant challenge as far as their anesthetic management is concerned. Most of these patients either take medicines or else are in a comparatively controlled state. Different protocols have been suggested for such cases such as corticosteroids, long-term β_2_ agonists, leukotriene receptor antagonists and theophylline sustained-release preparations (1).

Likewise, different anesthetic regimens have been forwarded to obviate any acute exacerbation of the asthmatic state which if evolves would undoubtedly bring in its wake a reduction in chest wall and lung compliance coupled with high peak airway pressures and a drastic reduction in oxygen saturation, alveolar arterial pressure and a concomitant increase in partial pressure of carbon dioxide. Such events eventually lead to either cancellation of surgery or else morbidity. During the last two decades, we have been routinely using promethazine 25 mg along with meperidine 50 mg intravenously as premedication in addition to fentanyl and midazolam immediately before the induction of anesthesia for all established but controlled cases of asthma. Using this protocol, we have never encountered any relapse of an asthmatic attack during surgery.

Promethazine, a member of the phenothiazine group of drugs is being used as a premedicant in anesthesia specially in those cases where an allergic component is forthcoming, because not only does this drug possesses sedative and hypnotic properties but at the same time has proven and obvious antihistaminic properties and anticholinergic effect (2–4). Again, it has been emphasized that promethazine possesses strong anticholinergic properties, blocking the responses to acetylcholine that are mediated by muscarinic receptors (4). Most of the opioids lead to bronchoconstriction and thus inevitably worsen pre-existing airway disease. It is an established fact that acetylcholine released by vagal stimulation induces smooth muscle contraction of the airways as it binds to the muscarinic receptors (5). Thus the cholinergic tone mediated through the vagal nerve can be substantially mitigated by using anticholinergic, and to serve this purpose meperidine stands unique in possessing both anticholinergic and analgesic effects. Our hypothesis is based on the valid assumption that promethazine is a bronchial dilator and meperidine has an atropine-like action.

It has been reported in the literature that meperidine could lead to histamine release and thus cause a reduction in systemic vascular resistance and blood pressure. However, this histamine releasing property attributed to meperidine can be prevented with the administration of H_1_ and H_2_ blockers (6). In our cocktail, we utilized both meperidine and promethazine in asthmatics. Promethazine in this combination could obviously prevent and offset the histamine releasing effect of meperidine owing to its H_1_ blocking effect and anticholinergic property. Meperidine in this combination exerted a meaningful vagolytic action and hence broncho-dilatation and the combined effects thus obtained provided significant improvement in the pulmonary function of asthmatic patients undergoing surgery. The vagolytic potential of meperidine as stressed upon in our interim report has also been unequivocally substantiated in other studies as well (7). Most of the opioids supposedly lead to bronchoconstriction and thus worsen preexisting airway disease as they are histamine releasers (8). Meperidine however stands unique because of its anticholinergic properties providing bronchiolar dilatation and a substantial reduction of bronchial secretions. Experimental data conducted also suggest that triggering some of the opioid receptors may in fact decrease bronchoconstriction. Bronchoconstriction caused by citric acid given to guinea pigs was decreased when parenteral and inhaled opioids were given (9). Although these data do not nullify the histamine releasing properties of opioids, nevertheless among the available opioids, meperidine has an anticholinergic property and on that basis appears to be of value in asthmatics by curtailing bronchial secretions and eliciting broncho-dilatation.

Thus this cocktail that we utilized in our patients could effectively help in counteracting an acute exacerbation of an asthmatic attack both intra and postoperatively. During our management of chronic asthmatics with this protocol, we have never witnessed any change in the lung compliance or an increase in peak-airway pressures. Although we have not conducted a randomized controlled trial in comparing this anesthetic regimen but are of the opinion that such a protocol of promethazine and meperidine if used in future studies would prove to be fruitful owing to the safe pharmacokinetic profile of the drugs.

We may conclude that as purported in the literature, meperidine possesses an atropine-like action or a vagolytic action thus facilitating broncho-dilatation. This broncho-dilatation coupled with the H_1_-blocking and anticholinergic effects of promethazine could safely stave off any broncho-constrictive effects as a result of meperidine owing to its histamine releasing property.
